# Citation Network Study on the Use of New Technologies in Neurorehabilitation

**DOI:** 10.3390/ijerph19010026

**Published:** 2021-12-21

**Authors:** Vanesa Abuín-Porras, Clara Martinez-Perez, Carlos Romero-Morales, Roberto Cano-de-la-Cuerda, Patricia Martín-Casas, Patricia Palomo-López, Miguel Ángel Sánchez-Tena

**Affiliations:** 1 Faculty of Sport Sciences, Universidad Europea de Madrid, 28670 Madrid, Spain; vanesa.abuin@universidadeuropea.es; 2 Fundación DACER, Área de I+D+I, San Sebastián de los Reyes, 28702 Madrid, Spain; 3 ISEC LISBOA—Instituto Superior de Educação e Ciências, 1750-179 Lisboa, Portugal; clara.perez@iseclisboa.pt (C.M.-P.); masancheztena@ucm.es (M.Á.S.-T.); 4 Department of Physiotherapy, Occupational Therapy, Rehabilitation and Physical Medicine, Faculty of Health Sciences, Rey Juan Carlos University, 28922 Madrid, Spain; roberto.cano@urjc.es; 5 Department of Radiology, Rehabilitation and Physiotherapy, Faculty of Nursing, Physiotherapy and Podiatry, Complutense University of Madrid, IdISSC, 28040 Madrid, Spain; pmcasas@enf.ucm.es; 6 University Center of Plasencia, University of Extremadura, 06006 Badajoz, Spain; patibiom@unex.es; 7 Department of Optometry and Vision, Faculty of Optics and Optometry, Universidad Complutense de Madrid, 28037 Madrid, Spain

**Keywords:** neurorehabilitation, citation network, CitNetExplorer, new technologies

## Abstract

New technologies in neurorehabilitation is a wide concept that intends to find solutions for individual and collective needs through technical systems. Analysis through citation networks is used to search scientific literature related to a specific topic. On the one hand, the main countries, institutions, and authors researching this topic have been identified, as well as their evolution over time. On the other hand, the links between the authors, the countries, and the topics under research have been analyzed. The publications analysis was performed through the Web of Science database using the search terms “new technolog*,” “neurorehabilitation,” “physical therapy*,” and “occupational therapy*.” The selected interval of publication was from 1992 to December 2020. The results were analyzed using CitNetExplorer software. After a Web of Science search, a total of 454 publications and 135 citation networks were found, 1992 being the first year of publication. An exponential increase was detected from the year 2009. The largest number was detected in 2020. The main areas are rehabilitation and neurosciences and neurology. The most cited article was from Perry et al. in 2007, with a citation index of 460. The analysis of the top 20 most cited articles shows that most approach the use of robotic devices and brain–computer interface systems. In conclusion, the main theme was found to be the use of robotic devices to address neuromuscular rehabilitation goals and brain–computer interfaces and their applications in neurorehabilitation.

## 1. Introduction

The term “neurological disorders” comprises a wide range of pathological situations, including both central and peripherical conditions of the nervous system, such as stroke, traumatic brain injury, multiple sclerosis, spinal cord injury, cerebral palsy, Parkinson disease, and many more. The prevalence of disability due to this conditions is increasing worldwide [1]. Therefore, there is a need to identify treatment strategies that address the multiple, complex impairments associated with neurological conditions.

The impact of neurodisability may include “difficulties with movement, cognition, hearing and vision, communication, emotion, and behavior” [2]. The term “neurorehabilitation” refers to the therapeutic strategies necessary to assess and provide health care for this range of conditions. Neurorehabilitation could be defined as a multidisciplinary and cross-sectorial approach that materializes from the merging of a group of related professional areas in the disability field [3].

According to the United States Office of Technology Assessment [4], medical technology is defined as a compilation of equipment, procedures, techniques, sanitary structures, information systems, and health care delivery practices that may be used by clinicians in prevention, diagnosis, treatment, and rehabilitation in order to improve any individual’s quality of life. New technologies in neurorehabilitation is a wide concept that intends to find solutions for individual and collective needs through the development of technical systems that contribute in a meaningful way to the acquisition and improvement of the motor, cognitive, and social abilities, directly related with daily living activities and active community participation, through an innovative attitude [5]. These technical systems allow to increase the number and duration of the therapy sessions, increase motivation and engagement, and facilitate precise records and evaluation [6]. The impact of new technologies in rehabilitation has led to the development of a wide range of assistive technologies that include electromyographic biofeedback, virtual reality, and electromechanical and robotic devices designed for therapy purposes, with the aim of reducing disability [7]. Some specific systems, such as virtual reality, specifically have a recognized impact in enhancing participation by allowing skill practice (motor or cognitive) in a highly motivating and engaging environment [8,9]. Moreover, there appears to be a substantial increase in attention in rehabilitation literature to such technologies.

Analysis through citation networks is used to search scientific literature related to a specific topic. Using this type of analysis, other additional relevant publications can be found with the aim of displaying in a qualitative and quantitative way the connections between papers and authors by means of the creation of groups [10]. Besides, it allows to quantify the most cited publications in each group to analyze the development of a specific research field or to focus the literature search on a specific subject [10,11]. This analysis is used to identify core research topics on a certain area of knowledge, main research groups, or leading institutions and the relations between them, facilitating the identification of opportunities for further advances in a given topic [12]. To the authors’ knowledge, no citation network studies have been conducted about new technologies applied in neurorehabilitation.

Consequently, considering the exponential increase in publications about new technologies in neurorehabilitation, the purpose of this study is to identify the current individual research areas and to determine the most cited publication. This would lead to a graphic conception and examination of the target interests in the field of new technologies in neurorehabilitation and the progression of institutions and research groups in different countries through time. Furthermore, this study intends to analyze relationships among publications and different research groups through Citation Net Explorer (CitNetExplorer) software (Ness Jan van Eck and Ludo Waltman, Centre for Science and Technology Studies (CWTS), Leiden University, Leiden, the Netherlands), which intends to facilitate the study of the evolution of scientific literature in a concrete research field.

## 2. Materials and Methods

### 2.1. Database

Exploratory research was conducted to select a database for performing the analysis. The publications research was performed through the Web of Science (WOS) and Scopus databases [13]. These databases provide essential information for bibliometric and citation network analysis, including title/abstract, keywords, number of citations, and author affiliations, which may not be available in other databases [14,15].

The terms used were “new technolog*,” “neurorehabilitation,” “physical therapy*,” and “occupational therapy*.” These terms were used in alignment with the purpose of this study, considering also that these are the most common terms used in all related research fields. Considering that the results of the searching showed a number of papers in common, the Boolean terms NOT and AND and the truncation symbol * were used. In this way, and through a manual review, we ensured that all the articles in this research area have been included but avoided their duplication, following the citation network methodology, which differs from systematic reviews in analyzing the connection between publications and not the publications per se [11]. In turn, the various keywords were combined to obtain research that contained interventions with new technologies. Thus, the terms used in the consecutive search operations were (1) (new technolog* AND “neurorehabilitation”); (2) (new technolog* AND neurological rehabilitation NOT “neurorehabilitation”); (3) (new technolog* AND “neuro-rehabilitation” NOT neurological rehabilitation NOT “neurorehabilitation”); (4) (new technolog* AND “physical therapy*” NOT neurological rehabilitation NOT “neurorehabilitation” NOT “neuro-rehabilitation”); and (5) (new technolog* AND “occupational therapy*” NOT neurological rehabilitation NOT “neurorehabilitation” NOT “neuro-rehabilitation” NOT “physical therapy*”).

On 27 December 2020, all original data were extracted from the online version of the Web of Science database using the “topic” method. The selected interval of publication was from 1992 to December 2020. With regard to the citation indexes, Social Sciences Citation Index, Science Citation Index Expanded and Emerging Sources Citation Index were used. To standardize the variations between authors and institutions citation forms, CiteSpace software (Chaomei Chen, College of Computing and Informatics, Drexel University, PA, USA) was used.

These databases were selected based on their relevance and use in the health field. The databases results were compared to determine the selection of the database for the research strategy. The exclusion criteria were articles without scientific relevance such as news, obituaries, projects, or patents, available in journals. Finally, the selected database was WOS, as it fitted the purpose of the study; it had a wide coverage on the topic and allowed the retrieval of the citation data, which is necessary for the analysis. WOS is widely used in meta-analysis-related studies, especially for researchers in China. Domains related to medical and health sciences and the traditional field of information sciences and librarianship stand out in the use of citation databases. In addition, it is the only base with extensive information in this field of research that allows citation networks to be carried out using various software [16,17]. The documents were downloaded in text format (.txt) for further analysis. The downloaded information contained total number of publications; names of the authors, their affiliations and the total number of articles, and the total citations of each author; most prolific countries and collaborations; institutional affiliations and frequency of citation; most cited articles with titles, authors, journal details, year of publication, total citations, and citations per year; and titles, summaries, and keywords.

### 2.2. Data Analysis

#### 2.2.1. Bibliometric Analysis

For the bibliometric analysis, a series of indicators was used to identify distributed characteristics and structural patterns of the bibliographic data. The annual number of publications of research results demonstrated the increasing trend of increasing publications on new technologies in neurorehabilitation. Journals with the highest number of publications were identified as those that published academic articles and contributed to the development of the research field. Both the journal impact factor (IF) and the quartiles in the relevant categories were derived from the Journal Citation Report (JCR; 2019) and SCImago Journal Rank (2019) and used to explore the influence of the editorial journal. Data from the quantitative analysis of the evolution of the literature, as well as bibliometric indicators, were used to present an informative overview of the research during the study period.

#### 2.2.2. Network Analysis

The results were analyzed using CitNetExplorer software (Ness Jan van Eck and Ludo Waltman, Centre for Science and Technology Studies (CWTS), Leiden University, Leiden, the Netherlands). This software allows to analyze and visualize citation networks between scientific publications. It also includes the possibility of managing millions of interconnected publications and citations. Therefore, a general network of several millions of publications can be initially handled to produce a more profound work of about a hundred publications on a predeterminate subject.

By means of the attribute “citation score,” a quantitative analysis of the most citated papers in a specific period of time was performed. This function allows to quantify internal connections in WOS and also external connections with other databases [11]. The “clustering” function is used to divide the publications into different groups regarding the connections among them, so that the most connected articles are generally in the same group attending to citation network criteria [11].

The function “identifying core publications” was used to point out which publications are considered as the nucleus of the network, considering they have a minimum number of connections with other central publications, and to eliminate nonimportant elements. This minimum number of connections is established by the research group. For the purpose of this study, following previously described criteria [11], the number of minimum connections was set in four. Finally, the function “drilling down” was used for a further, multilevel analysis of each group.

VOSviewer software (version 1.6.9, Centre for Science and Technology Studies, Leiden University, Leiden, the Netherlands) was used for obtaining figures. Thus, it was possible to carry out an in-depth network analysis to visualize the connections between the related elements and explain their network structure.

(a) Countries’ coauthorship network: The coauthorship analysis was carried out to identify the collaboration networks between the countries in this field of research. The nodes represent the countries participating in this field of research and the links between the elements implied cooperative relationships. As the number of articles published by an individual country increases, the size of the node increases in parallel. The number of links shows the number of times that a given country shared coauthorship with others. Thus, as the number of coauthors increases, the strength of the link increases.

(b) Cited references co-citation network: Nodes represent scientific references, and node size represents the number of times a reference is cited. The correlation of the articles according to the co-citation links was represented according to the distance between two references. Self-citations were not considered for the analysis.

(c) Author keyword co-occurrence network: Nodes represent the most frequently cited author keywords, and the size of an individual node represents how many times that keyword was cited. The strength of the link between two nodes indicates the number of articles in which two keywords appeared together [18].

Each group is determined by a resolution value, which ranges from 1.0 to 0.50 [18].

#### 2.2.3. Scientometric Analysis

In addition, for the scientometric analysis of the data, CiteSpace (5.6.R2; Chaomei Chen, College of Computing and Informatics, Drexel University, PA, USA) software was used. This Java-based tool links: (a) Kuhn’s model of scientific revolutions, which provides a fitting framework to derive our strategies for conducting our own systematic review; (b) Price’s scientific frontier theory, which describes the number of prolific authors in a subject field; (c) the organization of ideas; (d) the best information foraging theory of scientific communication; and (e) the theory of discrete and reorganized knowledge unit. This last theory is an approach to understanding how strategies and technologies for searching, gathering, and consuming information adapt to the flow of information in the environment [14,15]. Moreover, the scientometric analysis can also provide a specific evaluation using the H index, which is generally used to measure productivity and impact of research groups of different institutions. This index is calculated by evaluating the number of citations for certain papers published in a journal: h of the N published papers have been cited at least h times [16]. Another parameter—“degree”—shows the number of connections among authors, institutions, or countries in the “knowledge of co-occurrence” graph; the higher the degree, the greater the connection. “Intermediary centrality” measures the importance of nodes in the research cooperation network. Intermediary centrality is a measure of the number of times a node acts as a waypoint along the shortest path between two other nodes, geodetic distance. Finally, “half-life” is a parameter that represents the continuity of institutional research in time. It is defined as the number of years a post receives half of its citations since it was posted. A low citation half-life suggests citation activity that peaks and declines rapidly. A high cited half-life suggests citation activity that peaks and declines more slowly [14].

## 3. Results

### 3.1. Description of the Publications

The first articles were published in 1992; thus, the timeframe selected for the search was from 1992 to December 2020. A total of 454 publications and 135 citation networks were found according to title, abstract, and keywords. The most relevant publications were considered according to the number of citations.

The publications has exponentially increased from the year 2009 (1992–2008: 14.3%; 2009–2020: 85.7% publications). The year 2020 was the one with the largest number of publications—49—and one citation network. Out of all the found publications, 53.1% were articles, 25.7% were proceedings, 19.2% were reviews, 5.1% were editorial material, 4.1% were congress and conference summaries, and 2.7% were book chapters.

### 3.2. Characteristics of the Publications

**Figure 1:**
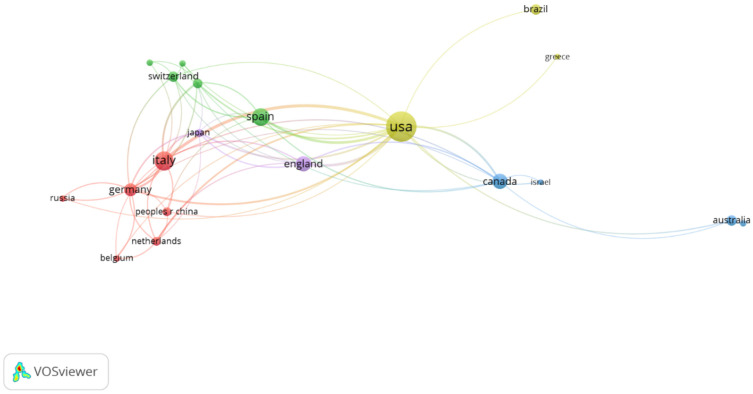
Collaboration between countries.

Regarding the publication’s language, 95.7% were written in English, 3.1% in Russian, and 2.2% in Spanish. As shown in Table 1 and Figure 1, countries with a larger number of publications were the United States (31.7%), Italy (13.0%), and Spain (11.0%). Figure 1 shows publications with most number of citations in the analysis as the group they belong to. The color of the article represents the group they belong to and the lines between elements represent bonds. In other words, each group has a different color, and the lines are the unions with other groups. We have explained it in the paper.

Supplementary Figure S1 shows the trajectory of the number of publications in the five countries with a largest number of papers about new technologies in neurorehabilitation in the past 10 years. The highest increment is shown in the United States. Besides, in the past 4 years, the number of publications in Italy has experienced an increment of 66.04%. The increase in the number of publications from countries such as the United Kingdom or the United States may be due to a number of different factors, including the fact that they are English-speaking countries or the possible affiliations that exist between different research groups in the scientific field community. It should be considered that in our study England stands out, without counting other countries in the United Kingdom.

According to the publication areas of the journals, research areas in the topic are multidisciplinary. The main areas are rehabilitation (29.8%) and neurosciences and neurology (27.3%) (Supplementary Table S1).

As shown in Supplementary Table S2 and Supplementary Figure S2, the authors with the largest number of publications are Iosa, M. (2.60%), Morone, G. (2.16%), and Paolucci, S. (2.16%). There are 18 clusters, where the most important is red with 43 authors, where the central author is Ardanza, A. The second most abundant group is green, composed of 41 authors, where the central author is Giovanni, M. The third group, in blue, is made up of 39 authors, led by the authors Amirabdollahian, F., and Loureiro R, C.V.

Institutions with a largest number of publications (Supplementary Table S3 and Supplementary Figure S3) are IRCCS Santa Lucia (1.76%), Universidad de Valencia (1.76%), and Consejo Superior de Investigaciones Científicas (CSIC; 1.32%). There are 15 clusters, where the most important is red with 23 institutions and the central institution is IRCCS Santa Lucia. The second most abundant group is the green one, made up of 21 institutions, where IRCCS Istituto Auxologico Italiano stands out. The third group, in blue, is made up of 18 institutions, led by the University of Alberta. In Supplementary Table S4 and Supplementary Figure S4, the main journals that have published on the topic and the number of publications are shown. There are 14 clusters, the most important of which are red and green, made up of 8 journals each, where *Journal of Neuroengineering and Rehabilitation* and *Plos One* stand out. The third group, in blue, is made up of seven journals, led by disability and rehabilitation assistive technology.

#### Keywords

**Figure 2:**
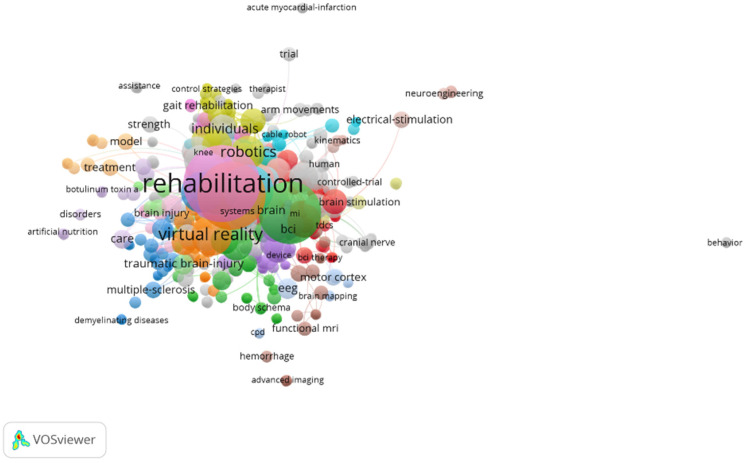

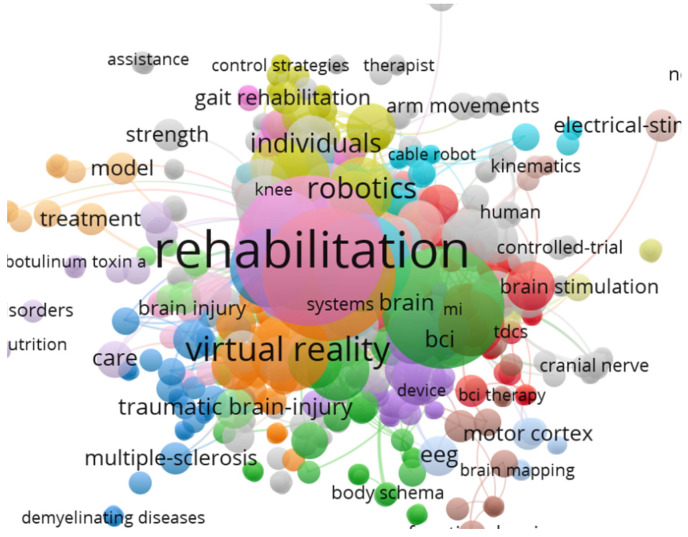
Connection between keywords.

Table 2 and Figure 2 show the keywords most frequent in the main publications.

In Table 3, the main details of the top five groups of Figure 2 are shown.

### 3.3. Most Cited Publications

The most cited article in this citation network was from Perry et al., published in 2007 with a citation index of 460. This study is based on the benefits of an exoskeleton applied to rehabilitation and virtual reality simulation. The authors used a pilot database in which the dynamics and kinematics of the arm performing daily live activities were established. This database was used for orientation to develop an “anthropomorphic, 7-DOF, powered arm exoskeleton.” In this article, the authors state that their model (CADEN)-7 offers versatility as an effective interface and stands as a new line of assistive technology with several applications, such as a tool for assessment and therapy in the physiotherapy area, a device to enhance regular human functions, haptic feedback for virtual reality simulators, and teleoperation.

The analysis of the top 20 most cited articles shows that 15 of them approach the use of robotic devices in neuromuscular rehabilitation and, moreover, the brain–computer interface systems and their applications in rehabilitation. Two of them deal with virtual reality technology in neurorehabilitation, other two are about benefits of robotic technology in subjects with walking disability, and, finally, one approaches the development of intelligent systems prototypes in motor rehabilitation and motor learning in subjects with neurological and neuropsychiatric conditions (Table 4).

### 3.4. Clustering

**Figure 3:**
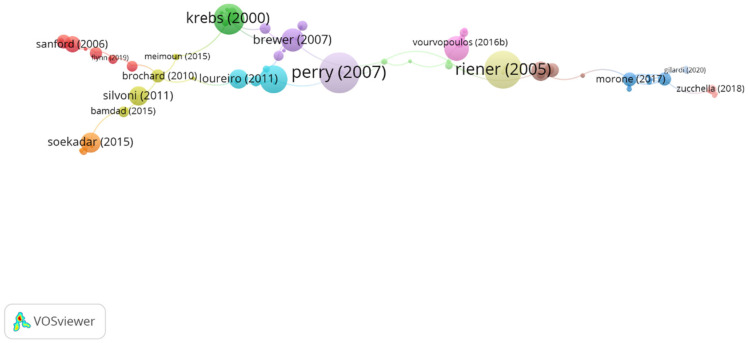
Clustering function in network citation about new technologies in neurorehabilitation. The most cited publication is that of Perry (2007).

Using the clustering function by CitNetExplorer, nine groups were found. All the publications found were divided into groups according to their connection to other publications, that is, how the authors cite each other. Five of them contain a significant group of articles, that is, more than five articles in each group and a resolution equal to 1, which means by having a high resolution power, the number of clusters will be greater. Nevertheless, the remaining four groups only reach 1.8% (*n* = 8 publications in total) (Figure 3). In Table 5, information about citation networks of the top five groups is displayed according to their size from the smallest to the largest group.

**Figure 4:**
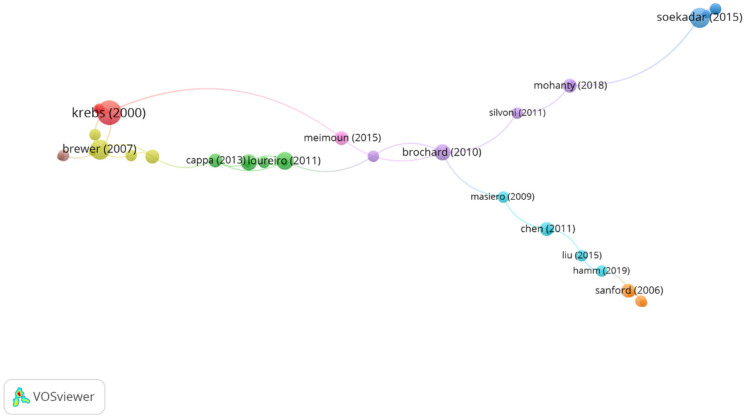
Group 1 citation network. The most cited publication is that of Krebs.

In group 1, denominated “robotic applications for upper limb,” 42 articles and 47 citations were found. The most cited publication was the article by Krebs et al. [19], published in 2000 in *Journal of Rehabilitation Research and Development*. In this paper, the authors intended to present their results regarding robot-aided therapy in 76 stroke patients. The research presents the clinical results not only regarding the effect of new technologies in the patient’s performance but also concerning the clinical importance of robotics as an effective tool to assess the outcomes of any intervention, pointing out that robot-based therapies have the advantage of being able to provide an exact report of the dosage of the therapy administrated and are capable to provide an accurate evaluation of their impact in the neuro-recovery process. They also provided future lines of practice evaluating the feasibility of different applications of these robotic devices, for instance, “classroom therapy” tools, in which a therapist would administrate the instructions for therapy for several patients at the same time, without disregarding the individual needs of each of them through the feedback provided by the robotic device. The articles of this group deal with techniques in robotic applications for upper limb therapy post stroke (Figure 4).

**Figure 5:**
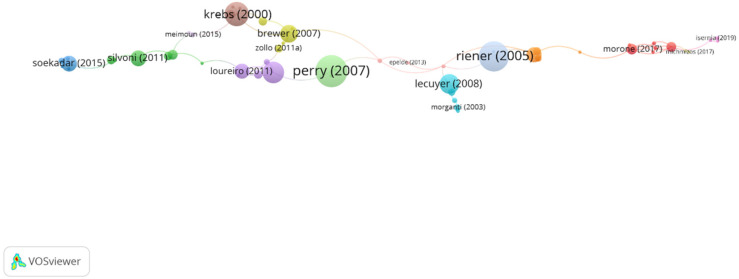
Group 2 citation network. The most cited publication is that of Perry (2007).

In group 2, denominated “robotic devices in neuromuscular rehabilitation,” 34 articles and 41 citations were found. The most cited publication is a paper by Perry et al. [17], published in 2006 in IEEE/ASME Transactions on Mechatronics, which also is the top one between the 20 most cited publications. The publications of this group aim to demonstrate that the use of robotic devices to approach the goals of neuromuscular rehabilitation represents a promising advance in medical care, such as the brain–computer interface systems and its applications in rehabilitation (Figure 5).

**Figure 6:**
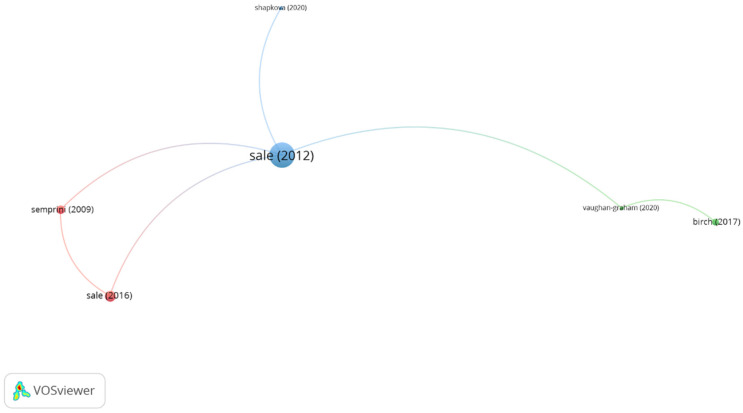
Group 3 citation network. The most cited publication is that of Sale (2012).

Concerning group 3, denominated “robotic technology in locomotor training,” 8 publications and 10 citations were found. The most cited publication is an article by Sale et al. [32], published in 2012 in *European Journal of Physical and Rehabilitation Medicine*. This paper deals with the use of robotic technology in subjects with walking disabilities, which has an enormous impact in activities of daily living, comprehending participation in social, vocational, and recreational events. The authors state that recent clinical practice may include the use of robotic device, that is, robot exoskeleton devices or end-effector devices to maximize the amount of practice without disregarding the individual clinical aims. This article presents two robotic aids, the ReWalk (Argo Medical Technologies Ltd. USA), which consists of an external mechanism with joints and links imitating the human body and the end-effector-based robot, which consists of movable footplates attached to the patient’s feet and a system of body suspension. Sale et al. stated that the goal of this techniques is to develop devices that can be used by therapist and patients easily to enhance the efficacy of therapies. The publications in this group focus on how robotic technology allows an intensive locomotor training using normal gait patterns in the lower limb in patients with walking disabilities (Figure 6).

**Figure 7:**
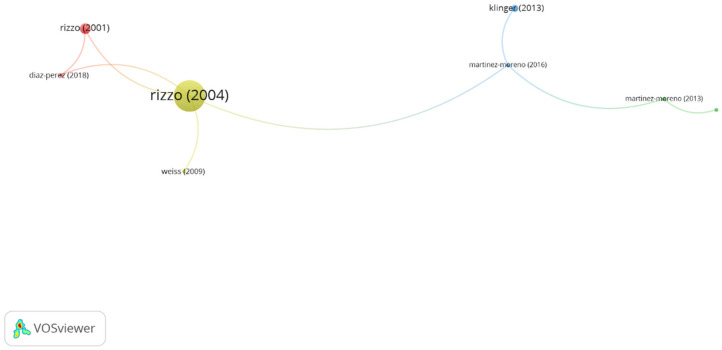
Group 4 citation network. The most cited publication is that of Rizzo (20004).

In group 4, denominated “virtual reality,” 8 publications and 8 citations were found. The most cited publication is an article from Rizzo et al. [21], published in 2008 in *Neuropsychological Rehabilitation*. This paper focuses on the neuropsychological applications of virtual reality devices. The authors state that virtual reality allows accurate control of 3D environments providing precise, dynamic, multisensory stimulation. Besides, this technology allows the record of the patient’s behavioral responses. This offers a framework for several virtual reality devices that are detailed in the article. The authors conclude that the combination of functionally relevant virtual environments and a set of virtual reality assets may be of use in the body of knowledge about how cognitive and functional behavior are evaluated and rehabilitated. The publications of this group emphasize the value of virtual reality and the opportunities that its use offers in the development of new assessment and rehabilitation tools in neuropsychological rehabilitation, and the use of videogames in therapy for children affected with cerebral palsy (Figure 7).

**Figure 8:**
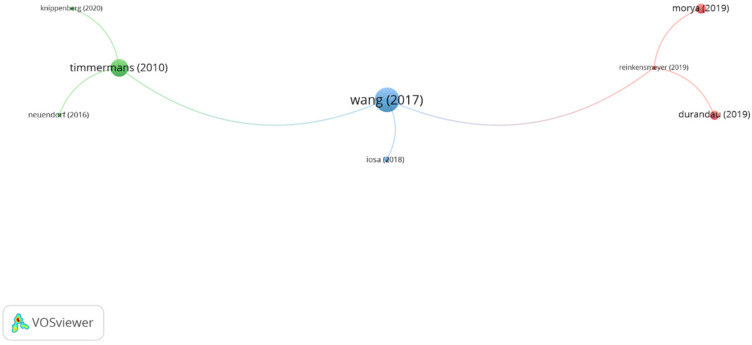
Group 5 citation network. The most cited publication is that of Timmermans (2010).

In group 5, denominated “intelligent systems,” 8 publications and 7 citations were found. The most cited article was the publication by Timmermans et al. [37], published in 2010 in *IEEE/ASME Transactions on Mechatronics*. The aim of the study was to evaluate the effects of a device designed for upper limb therapy, named T-TOAT. The device includes movement-tracking sensors, a set of task-oriented exercises, and a software-based toolkit. The authors concluded that the training with the T-TOAT is feasible, and patients improve their arm and hand performance and health-related quality of life significatively. The publications of this group focus on the development of intelligent systems to assist motor rehabilitation and motor learning in subjects with neurological and neuropsychiatric conditions (Figure 8).

After analyzing the connection between groups, no links were found among them. Therefore, each group focuses on definitely different topics.

### 3.5. Core Publications

**Figure 9:**
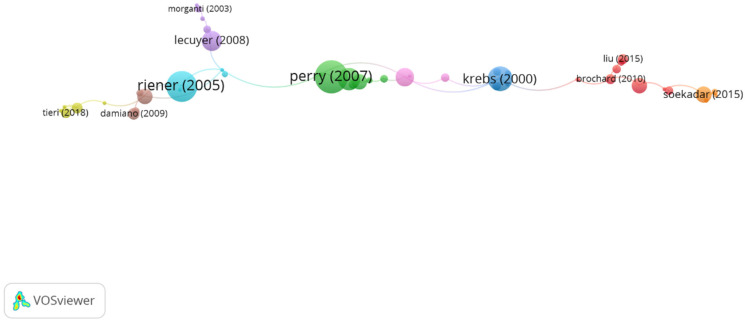
Core publications in the citation network about new technologies in neurorehabilitation.

A total of 238 publications with four or more citations were found, representing 52.4% of the publications, and 75 network citations were obtained. Consequently, these data from the network analysis demonstrate that the research on this field is a growing area. That is, given the high percentage of publications with four or more citations with respect to the publications found, it indicates that this research topic is frequently analyzed and cited. In this analysis, the main topic is the use of robotic devices to approach neuromuscular rehabilitation goals and the brain–computer interfaces and their applications in neurorehabilitation (Figure 9). At the same time, it shows that new groups present more difficulties to enter the field.

## 4. Discussion

Bibliometric analysis through citation network is still a relatively novel quantitative method of evaluation of the impact of academic research. It also allows the examination of relationships among research groups, institutions, and even countries. Overall, citation network analysis provides a clear diagram of the most cited publications in a research area [12]. The analysis of new technologies in neurorehabilitation under this focus could be helpful for many researchers in identifying collaboration chances between colleagues, multidisciplinary possibilities of teamwork, and creation of new groups with an international vision [38].

The aim of this study is to facilitate the identification of areas that can be considered a “pool” of opportunities and interesting networking with colleagues from other institutions and countries. Analysis of the relationships among publications and different research groups was performed through Citation Net Explorer (CitNetExplorer) software using the WOS database. As a result, the main research areas related to new technologies applied in neurorehabilitation were identified and the most cited publication was determined. Certain databases such as WOS or Scopus allow the construction of citation networks. Scopus includes a greater variety of publications compared to PubMed and WOS. On the other hand, the citation analysis that WOS presents offers better graphics and is more detailed than the citation analysis performed by Scopus. This is probably because WOS was done for the purpose of citation analysis. Regarding PubMed, it focuses on the clinical and biomedical literature, and Web of Science is interdisciplinary and includes the highest quality journals in each subject area [11].

However, the utility of some of these is limited to performing a systematic review of the literature, as they fail to offer a general vision of the connections between citations in a group of publications. This is the main reason for the use of CitNetExplorer and CiteSpace software in this study, as they allow to visualize, analyze, and explore citation networks in scientific publications [11]. On the other hand, the WOS database is one of the widest databases, beginning its search frame in the year 1900. Nevertheless, as a limitation of this study, WOS only accepts international journals and their selection process is exhaustive, meaning that some publications that are not in WOS could have been excluded in this analysis. Concerning journals with a particularly large number of publications about new technologies in neurorehabilitation, *Journal of Neuroengineering and Rehabilitation* stands out, occupying the 11th place in health informatics and 6th place in rehabilitation. Moreover, this journal shows the highest impact factor, 3.52. Nevertheless, it is significant to ponder that, although the impact factor is a critical index, it is not an absolute index, as the impact factor does not take into account the impact of the research results, as well as the authors’ physical and intellectual contributions [39].

Forty-five of the publications were included in journals contained within the category “rehabilitation,” which comprises clinical specialties with strong connections with neurological recovery, such as physical therapy, occupational therapy, neuropsychology, and rehabilitation medicine. The countries with a higher number of publications that were detected in this study were the United States, Italy, and Spain. There might be multiple reasons associated with this fact. High prevalence of neurological conditions in these countries, presence of specialized institutions that may attract public and private funding and technological-centered research investment, among others, could be plausible explanations for this finding. In addition, the high numbers of publications from countries such as the United States has been linked to the fact that it is an English-speaking country and most journals use English as publishing language or connections between research groups within the scientific community [40,41].

Citation network evolution through the years is intrinsically connected to the historical context of research in each decade. The development of rehabilitation robots started in the late decade of 1980. The next period was a pioneer phase [42]. From the year 2000, the first commercially available robots appeared [43]. The research literature experiments a shift with the apparition of publications regarding the analysis of the potential use of videogames in therapy. In 2001, Prensky concluded that playing videogames provides not only conceptual procedures learning but also skill practice that leads to a process of internalization [44]. Therefore, a successful game design could enhance significatively not only the skill acquisition during the therapy session but also long-term learning [45]. A few years later, studies by Riener et al. [18] and Lünenburger et al. [28] (2005, 2007) analyzed the effect of biofeedback in robot-assisted gait rehabilitation. Their conclusions were that robot-assisted gait combined with either feedback provided by the robotic system or “patient-cooperative” adaptations could increase the length of the therapy session and reduce the physical tension for the therapist in the next years, these two areas—robotic and videogames—seem to converge. In 2008, Lécuyer et al. [23] analyzed the connections between brain–computer interfaces (BCI), videogames, and virtual reality technologies. They discussed in their study the new findings in the time of the publication: devices that allowed people to navigate virtual environments or manipulate virtual objects using brain activity exclusively. The authors established as future lines for new technologies the development of lines of research, including basic neurosciences, optimize peripheric devices, mental gamepads, the creation of more effective signal-processing techniques and the invention of new adaptative interaction paradigms and innovation in gaming. This highly cited study foretold the path that research involving new technologies applications for therapy was going to follow in the next years.

In the next decade, from 2009 to 2019, the growing interest on the area leads to a significative increase in publications. One of the most cited publications of that frame time is the article by Soekadar et al. [25], in which BCI development is analyzed. As previously mentioned, BCI seem to be, in 2008, one of the main research areas in this topic according to Lécuyer et al. BCI translate cerebral activity to computer control signals or external devices control signals. In this paper, two new strategies to overcome motor paralysis related to stroke are offered. The first strategy is related to the ability of BCI to establish continuous high-dimensional brain control of robotic devices or functional electric stimulation. The second strategy is related to the facilitation of neuroplasticity processes, increasing the possibility of motor learning and rehabilitation. It has to be pointed out that, during this decade, motor learning research was also showing an exponential increase [46], which could explain the fact that institutions and groups explored the applications of BCI and robotics to that field. The authors state that BCI systems would increase their role in post-stroke therapy. Two years later from the study by Soekadar, Morone et al. [47] also published a highly cited brief review of robotic gait assistance devices, including several commercial models, pointing out them as an important emerging field. The authors concluded that, even though the robotic gait systems can improve the therapy results, it is important that neuroscientific principles are used to guide interventions, which is consistent with the interest in motor learning processes that marked that decade [46].

The identified “key year” due to its high number of publications and the development of this research field is year 2020. Among the published articles, the paper by Pavon et al. [48] stands out in citation number. The article analyzes the impact of hospital-acquired disability through a portable technological device, an accelerometer attached to the patient’s ankle. The authors conclude that portable technology data can be used to indicate the need for physical/occupational therapy in certain patients. Moreover, in the same year, another highly cited article was a systematic review carried out by a research group from Italy, Matamala-Gómez et al. [49], in which the focus is placed in telerehabilitation systems in patients with neurological pathology. Concretely, they analyzed the engagement strategies implied. The identified strategies in the reviewed publications were mainly patient self-management and self-awareness, patient motivation, and patient adherence subcomponents of engagement. The authors concluded that the future lines of research would have to include designs that comprise both qualitative and quantitative measures of telerehabilitation programs regarding patient engagement in neurorehabilitation through this new technological approach. The importance of telerehabilitation in the year 2020 due to the impact of the COVID-19 pandemic could be an explanation for the high number of citations of papers that address systems of telemonitoring and data collections. A citation network study from Martínez-Pérez et al. (2020) identified 14,335 publications and 42,374 citations regarding COVID-19 [50]. The care model was forced to change dramatically in a really short period of time. This could explain the high interest in publications related to technologies that allow clinicians to maintain their therapeutic interventions and evaluations in the pandemic context.

It is important to contemplate some emerging fields that, despite being still under the established threshold of citation network for this study, are developed exponentially and are considered solid future lines for the area of new technologies research and publishing. Mobile technology and its use by patients and professionals in prevention, assessment, treatment, or monitoring of the health status in neurorehabilitation has enormously increased its importance in the last few years [51]. The term mHealth is defined as the use of mobile applications in the health field, including diagnosis and decision-making, but that is not a standardized definition yet [52]. mHealth shows a great potential to reduce healthcare costs, increasing the achievement of positive therapy results in neurological conditions that imply rehabilitation interventions by offering the possibility of continuous monitoring, promoting healthy lifestyle, reducing the need of medical visits, and improving the quality of the healthcare services regardless of time or distance.

Moreover, the final goal of new technologies in neurorehabilitation is to improve functional independence in the patients; consequently, future lines of research should approach some challenges as home implementation including the possibility of distant monitoring (telerehabilitation) and, primarily, cost reduction. Regarding this, augmented reality and mixed augmented reality-based technologies are taking a prominent lead. Nevertheless, their exploitation and scientific bases are still underdeveloped. Finally, future lines should also include domotic and intelligent environments as part of their research studies in this populations [5].

Regarding the limitations of this study, it should be noted that only Web of Science documents were used for data analysis. However, as previously specified, the bibliographic search has been carried out in other databases, ensuring that the entire bibliography has been included. In addition, research from conference venues has not been included. Although unpublished work from conferences can be striking and promising for a field, it is not until this work is peer reviewed, published, and cited that it can enter a network citation analysis. The purpose of this kind of analysis is to identify opportunities worldwide as well as research areas and leading groups in a specific field and are used in many areas to provide a research target for investigation. Doubtless, with time, information from conference venues can potentially become a main research target, but it cannot be stated in that very moment, it depends on many factors that are analyzed in citation network analysis (i.e., funding from institutions, countries, collaboration between groups).

## 5. Conclusions

This study accomplished an objective visualization and analysis of the research interests and achievements in the field of new technologies in neurorehabilitation and the evolution of countries, institutions, and research groups through time. The citation network showed the increasing interest in robotic technology and brain–computer interfaces applied to neurorehabilitation and an exponential growth of the area in 2020, with many citations in publications involving telerehabilitation systems. This could be explained by the impact of the pandemic situation in the clinical care model. Some emerging areas, such as mobile applications in neurorehabilitation, are still under the established threshold of citation.

## Figures and Tables

**Table 1:** Countries with the largest number of publications.

Country	Publications (%)	Centrality	Degree	HalfLife
United States	144 (31.7%)	0.19	18	22.5
Italy	59 (13.00%)	0.12	15	12.5
Spain	50 (11.0%)	0.11	12	8.5
Canada	37 (8.1%)	0.10	11	10.5
England	36 (7.9%)	0.34	20	16.5

**Table 2:** Top 30 most frequent keywords.

Keyword	Frequency	Centrality	Degree	Total Link Strength
Rehabilitation	41	0.32	54	878
Neurorehabilitation	38	0.53	63	594
Stroke	29	0.24	41	718
Virtual reality	19	0.21	37	242
Brain–computer interface	11	0.08	21	137
Spinal cord injury	10	0.07	17	143
Exoskeleton	9	0.07	17	95
Assistive technology	7	0.05	15	88
Gait	7	0.03	15	158
Robotics	6	0.07	11	149
Motor learning	6	0.06	20	133
Neuroplasticity	6	0.05	16	116
Electroencephalogram	5	0.03	10	65
Cerebral palsy	5	0.03	10	72
Cognitive rehabilitation	4	0.06	12	76
Telerehabilitation	4	0.02	9	107
Balance	3	0.03	10	163
Motor imagery	3	0.01	8	91
Noninvasive brain stimulation	3	0.04	6	88
Technology	3	0.04	7	201
Brain injury	3	0.05	12	44
Brain stimulation	3	0.01	11	45
Interactive video	3	0.01	7	18
Gait rehabilitation	3	0.03	7	43
Eye-tracking	3	0.01	7	18
Neurological disease	3	0.04	9	30
Serious game	2	0.00	4	9
Cueing	2	0.03	6	18
Electrical stimulation	2	0.02	6	35
Brain plasticity	2	0.04	6	68

**Table 3:** Details of most frequent keywords.

Cluster	Color	Main Keywords	Topic	%
1	Red	Motor learning, transcranial magnetic stimulation, noninvasive brain stimulation, cortical reorganization, motor rehabilitation	Motor rehabilitation and motor learning	6.00
2	Green	Neurorehabilitation, performance, environments, randomized controlled trial, upper limb	Upper limb neurorehabilitation	5.60
3	Dark blue	Feasibility, efficacy, cognitive impairment, multiple sclerosis, dysfunction	Neurodegenerative pathology neurorehabilitation	5.60
4	Yellow	Walking, robotics, robot, locomotion, body weight support	Robotic technologies in subjects with walking disabilities	5.10
5	Purple	Communication, motor imagery, brain–computer interface, functional electrical stimulation, brain–computer interface	Brain–computer interface and applications to neurorehabilitation	5.00

**Table 4:** Top 20 most cited papers.

Author	Title	Journal	Year	Citation Index	Links
Perry et al. [17]	Upper-limb powered exoskeleton design	*IEEE/ASME Transactions on Mechatronics. 2007 Aug;12(4):408–417*	2007	467	4
Riener et al. [18]	Patient-cooperative strategies for robot-aided treadmill training: first experimental results	*IEEE Trans Neural Syst Rehabil Eng. 2005 Sep;13(3):380–94*	2005	402	4
Krebs et al. [19]	Increasing productivity and quality of care: robot-aided neuro-rehabilitation	*l Res Dev. Nov-Dec 2000;37(6):639–52*	2000	266	7
Loureiro et al. [20]	Upper limb robot mediated stroke therapy: GENTLE/s approach	*Autonomous Robots. 2003 Jul;15:35–51*	2003	212	4
Rizzo et al. [21]	Analysis of assets for virtual reality applications in neuropsychology	*Neuropsychol Rehabil. 2004 Jan;14:207–239*	2004	199	4
Wilson et al. [22]	Advances in electronic-nose technologies developed for biomedical applications	*Sensors (Basel). 2011;11(1):1105–76*	2011	191	0
Lécuyer et al. [23]	Brain–computer Interfaces, virtual reality, and videogames	*IEEE/ASME Transactions on Mechatronics. 2008 Oct;41(10):66–72*	2008	170	2
Brewer et al. [24]	Poststroke upper extremity rehabilitation: a review of robotic systems and clinical results	*Top Stroke Rehabil. 2007 Dec;14(6):22–44*	2007	147	7
Soekadar et al. [25]	Brain–machine interfaces in neurorehabilitation of stroke	*Neurobiol Dis. 2015 Nov;83:172–9*	2015	112	6
Silvoni et al. [26]	Brain–computer interface in stroke: a review of progress	*Clin EEG Neurosci. 2011 Oct;42(4):245–52*	2011	108	2
Loureiro et al. [27]	Advances in upper limb stroke rehabilitation: a technology push	*Med Biol Eng Comput. 2011 Oct;49(10):1103–18*	2011	107	5
Lüenenburger et al. [28]	Biofeedback for robotic gait rehabilitation	*J Neuroeng Rehabil. 2007 Jan 23;4:1*	2007	105	5
Acevedo et al. [29]	Nonpharmacological cognitive interventions in aging and dementia	*J Geriatr Psychiatry Neurol. 2007 Dec;20(4):239–49*	2007	106	0
MacPhee et al. [30]	Wheelchair skills training program: a randomized clinical trial of wheelchair users undergoing initial rehabilitation	*Arch Phys Med Rehabil. 2004 Jan;85(1):41–50*	2004	101	0
Jackson et al. [31]	Neural interfaces for the brain and spinal cord: restoring motor function	*Nat Rev Neurol. 2012 Dec;8(12):690–9.*	2012	93	0
Sale et al. [32]	Use of the robot-assisted gait therapy in rehabilitation of patients with stroke and spinal cord injury	*Eur J Phys Rehabil Med. 2012 Mar;48(1):111–21*	2012	91	4
Manhal-Baugus [33]	E-therapy: practical, ethical, and legal issues	*Cyberpsychol Behav. 2001 Oct;4(5):551–63.*	2001	85	1
Carbonaro et al. [34]	Integration of e-learning technologies in an interprofessional health science course	*Med Teach. 2008 Feb;30(1):25–33.*	2008	84	1
Sanford et al. [35]	The effects of in-home rehabilitation on task self-efficacy in mobility-impaired adults: a randomized clinical trial	*J Am Geriatr Soc. 2006 Nov;54(11):1641–8.*	2006	74	3
Padovani et al. [36]	Neurocognitive function after radiotherapy for paediatric brain tumours	*Nat Rev Neurol. 2012 Oct;8(10):578–88*	2012	73	0

**Table 5:** Information about the citation network of the top five groups.

Main Cluster	Number of Publications	Number of Citation Links	Number of Citations Median (Range)	Number of Publications with ≥4 Citations	Number of Publications in 50 Most Cited Publication
Group 1	42	47	16 (0–266)	29	12
Group 2	34	41	8 (0–467)	24	7
Group 3	8	10	17 (0–93)	6	1
Group 4	8	8	2 (0–199)	3	1
Group 5	8	7	9 (0–72)	6	1

## Data Availability

Not applicable.

## Supplementary Materials

The following are available online at https://www.mdpi.com/article/10.3390/ijerph19010026/s1, Supplementary Table S1. Top 10 research areas with the highest number of publications; Supplementary Table S2. Top 10 authors with the largest number of publications; Supplementary Table S3. Top 10 institutions with the largest number of publications; Supplementary Table S4. Top ten journals with the largest number of publications; Supplementary Figure S1. Number of publications by country and year; Supplementary Figure S2. Collaboration between authors; Supplementary Figure S3. Collaboration between institutions; Supplementary Figure S4. Collaboration between journals.
