# Supplementary material for: Citation Network Study on the Use of New Technologies in Neurorehabilitation
Source: Int J Environ Res Public Health. 2021 Dec 21;19(1):26. doi: 10.3390/ijerph19010026 (PMC8751120; doi:10.3390/ijerph19010026)
Supplement: Supplementary file 1 [file ijerph-19-00026-s001.zip › ijerph-1400622-supplementary.pdf]

Supplementary Material.

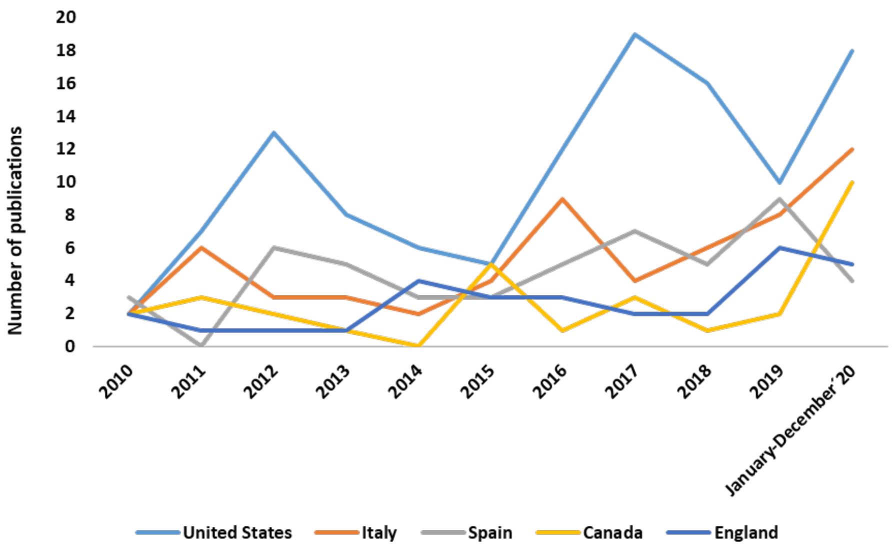

Supplementary figure S1. Number of publications by country and year.

.

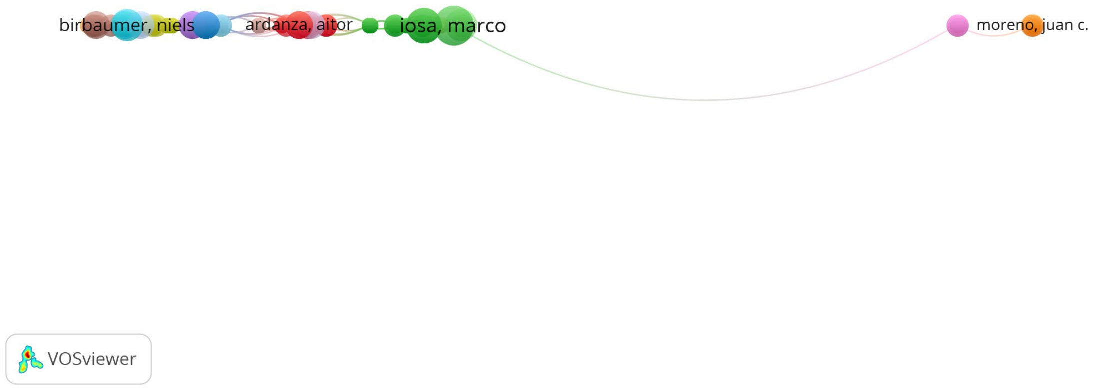

Supplementary figure S2. Collaboration between authors.

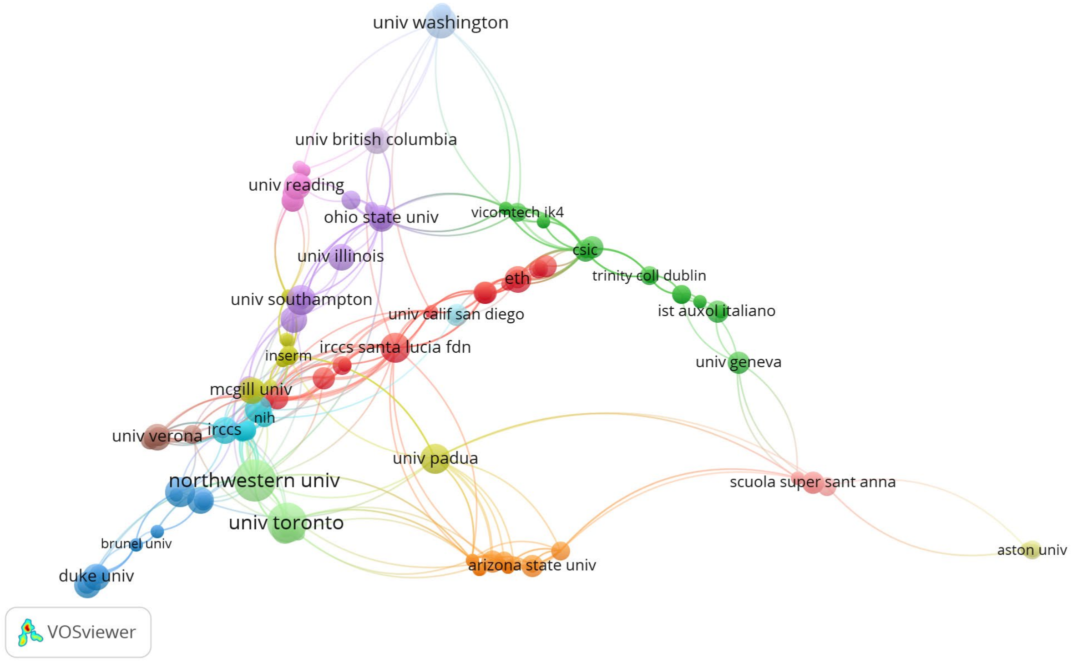

Supplementary figure S3. Collaboration between institutions.

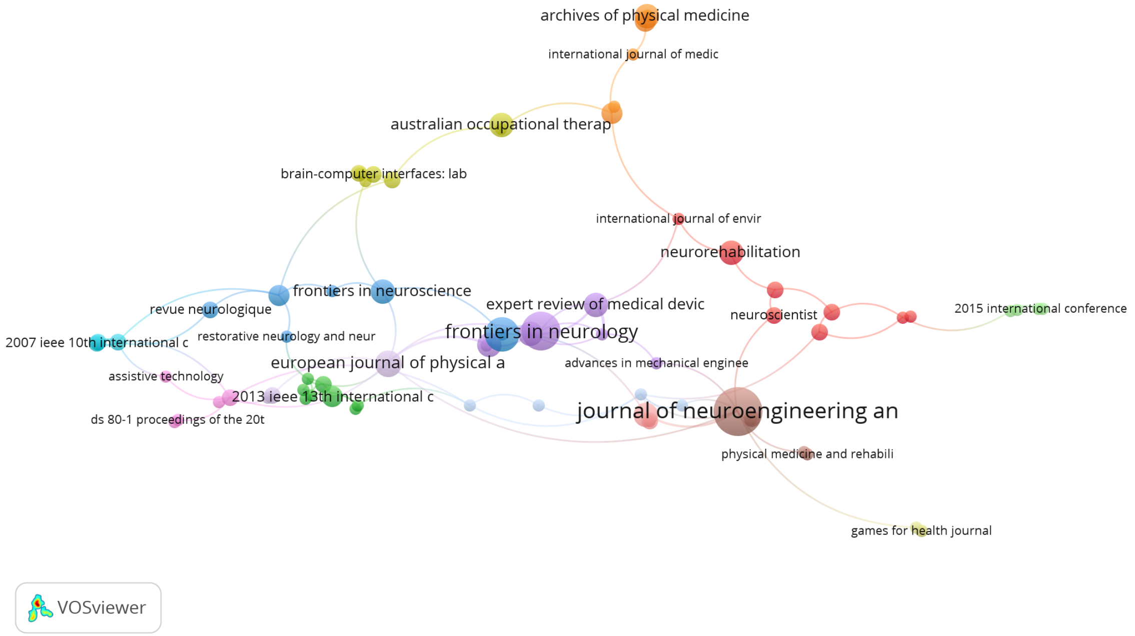

Supplementary figure S4. Collaboration between journals.

Supplementary table S1. Top 10 research areas with the highest number of publications

Category	Frequency	Centrality	Degree	HalfLife
Rehabilitation	132	0.17	37	22.5
Neurosciences and Neurology	124	0.32	48	21.5
Clinical Neurology	71	0.08	29	19.5
Biomedical Engenieering	70	0.12	44	14.5
Computer Sciences	58	0.12	45	14.5
Robotics	29	0.01	21	12.5
Electric and Electronic Engenieering	27	0.06	31	15.5
Medical Information	23	0.01	13	7.5
Internal and General Medicine	20	0.06	17	18.5
Psychology	19	0.11	19	2.5

Supplementary table S2. Top 10 authors with the largest number of publications

Author	Number of publications	*H* Index	Total citations	Citation average*	Centrality	Degree
Iosa M	6	4	115	19.5	0.00	6
Morone G	5	4	113	22.6	0.00	4
Paolucci S	5	4	113	22.6	0.00	4
Casana J	4	0	0	0	0.00	6
Nygard L	4	4	53	13.25	0.00	3
Volpe BT	3	3	305	101.67	0.00	5
Ardanza A	3	2	20	6.67	0.00	11
De Mauro A	3	2	20	6.67	0.00	11
Gomez EJ	3	1	2	0.67	0.00	6
Hummer FC	3	3	63	21.0	0.00	0

* Self-citations have not been considered

Supplementary table S3. Top 10 institutions with the largest number of publications

Category	Frequency	Centrality	Degree	HalfLife
IRCCS Santa Lucia	8	0.00	2	2.5
Universidad de Valencia	8	0.00	1	1.5
Consejo superior de investigaciones científicas CSIC	6	0.00	1	0.5
State University system of Florida	5	0.00	0	-0.5
Universidade de Sao Paulo	5	0.00	0	-0.5
Northwestern university	4	0.02	16	2.5
University of Montreal	4	0.00	2	-0.5
Catholic University of The Sacred Heart	4	0.06	5	-0.5
Sapienza university Rome	4	0.00	2	2.5
University of california system	4	0.00	3	-0.5

Supplementary table S4. Top ten journals with the largest number of publications

Journal	Total number of publicacions	Impact factor (2019)	Quartile	SJR (2019)	Citation/Docs (2 years)	Total number of citations (2019)	Centrality	*H* Index	Country
Journal of neuroengineering and rehabilitation	15	3.52	Q1	1.14	4.228	1652	0.00	85	United Kingdom
Disability and rehabilitation assistive technology	11	-	-	0.56	2.221	652	0.00	35	United Kingdom
Frontiers in neurology	9	2.89	Q2	1.06	3.184	6086	0.00	57	Switzerland
British journal of occupational therapy	8	0.96	Q3	0.38	1.007	253	0.00	44	United Kingdom
American journal of occupational therapy	6	2.23	Q1	0.71	1.373	607	0.00	77	United States
Functional neurology	5	1.85	Q3	0.72	1.700	184	0.00	40	Italy
Scandinavian journal of occupational therapy	5	1.35	Q2	0.50	1.608	228	0.00	37	United Kingdom
Physical Therapy	4	3.14	Q1	1.14	2.990	1355	0.00	142	United States
European journal of physical and rehabilitation medicine	3	2.26	Q1	0.83	2.420	717	0.00	53	Italy
Expert review of medical devices	3	2.20	Q3	0.60	2.388	726	0.00	60	United Kingdom
